# *OsVIL2* Regulates Spikelet Development by Controlling Regulatory Genes in *Oryza sativa*

**DOI:** 10.3389/fpls.2018.00102

**Published:** 2018-02-06

**Authors:** Hyeryung Yoon, Jungil Yang, Wanqi Liang, Dabing Zhang, Gynheung An

**Affiliations:** ^1^Graduate School of Biotechnology and Crop Biotech Institute, Kyung Hee University, Yongin, South Korea; ^2^Joint International Research Laboratory of Metabolic and Developmental Sciences, Shanghai Jiao Tong University–University of Adelaide Joint Centre for Agriculture and Health, School of Life Sciences and Biotechnology, Shanghai Jiao Tong University, Shanghai, China

**Keywords:** *VIN3-LIKE* gene, spikelet development, floral organ number, rice, Polycomb repressive complex 2, chromatin remodeling factor

## Abstract

Flower organ patterning is accomplished by spatial and temporal functioning of various regulatory genes. We previously reported that *Oryza sativa VIN3-LIKE 2* (*OsVIL2*) induces flowering by mediating the trimethylation of Histone H3 on *LFL1* chromatin. In this study, we report that *OsVIL2* also plays crucial roles during spikelet development. Two independent lines of T-DNA insertional mutants in the gene displayed altered organ numbers and abnormal morphology in all spikelet organs. Scanning electron microscopy showed that *osvil2* affected organ primordia formation during early spikelet development. Expression analysis revealed that *OsVIL2* is expressed in all stages of the spikelet developmental. Transcriptome analysis of developing spikelets revealed that several regulatory genes involved in that process and the formation of floral organs were down-regulated in *osvil2*. These results suggest that *OsVIL*2 is required for proper expression of the regulatory genes that control floral organ number and morphology.

## Introduction

Grasses have a unique inflorescence unit, the spikelet, that contains a different number of florets and glumes depending on species (Bommert et al., 2005). The spikelet of rice (*Oryza sativa*) consists of two rudimentary glumes, two empty glumes, and a single floret (Bommert et al., 2005). Each floret is composed of a lemma, a palea, two lodicules on the palea side, six stamens, and a carpel (Yoshida and Nagato, 2011).

APETALA2/ethylene responsive factor (AP2/ERF) family genes, *SUPERNUMERARY BRACT* (*SNB*), and *INDETERMINATE SPIKELET1* (*IDS1*), play crucial roles in the transition from spikelet meristem (SM) to floral meristem (FM) (Lee et al., 2007; Lee and An, 2012). Their mutant lines produce repetitive glumes and show an abnormal floral organ pattern due to extended activity of SM. Another AP2/ERF gene, *MULTI-FLORET SPIKELET1* (*MFS1*), also has a role in regulating SM fate. In *mfs1* mutants, additional lemma-like organs and elongated rachilla are produced, and empty glumes and palea are degenerated (Ren et al., 2013). These results suggest that proper transition from SM to FM is needed for normal spikelet development.

Analysis of various rice mutants has revealed several genes involved in glume development. For example, mutations in *EXTRA GLUME1* (*EG1*) and *EG2*/*OsJAZ1*, which function in jasmonic acid signaling, cause abnormal spikelet phenotypes. Empty glumes are transformed into lemma-like organs and extra glumes are produced (Li et al., 2009; Cai et al., 2014). In addition, floral organ identity and number are affected. These changes are probably due to altered expression of *OsMADS1* in the mutants (Jeon et al., 2000a; Prasada et al., 2005). Similar phenotypes are observed for rice *INDETERMINATE GAMETOPHYTE1* (*OsIG1*) RNAi plants (Zhang et al., 2015). Mutations in *long sterile lemma* (*G1*) are associated with homeotic transformation of the sterile lemma to a lemma, suggesting that the gene represses lemma identity to specify sterile lemma (Yoshida et al., 2009). Mutations of *OsMADS34* cause pleiotropic effects including alteration of empty glumes into lemma-like organs (Gao et al., 2010).

The development of palea is retarded in mutants defective in *RETARDED PALEA1* (*REP1*) (Yuan et al., 2009). *DEPRESSED PALEA 1* (*DP1*), encoding AT-hook DNA binding protein, also plays a crucial role in palea development (Jin et al., 2011). Mutations of that gene cause a palea defect as well as an increase in floral organ numbers. Expression analyses have indicated that *DP1* functions upstream of *REP1*. Mutations of *OsMADS15* also result in defective palea (Wang et al., 2010), while those of *OsMADS6* also have disturbed palea and altered carpel development (Ohmori et al., 2009; Li et al., 2010). Mutations of *OsMADS32* are linked with defective marginal regions for palea and ectopic floral organs (Sang et al., 2012).

Polycomb group proteins (PcG) are epigenetic repressors that control gene expression (Mozgova and Hennig, 2015). They function in various developmental processes by forming Polycomb repressive complex 2 (PRC2), which inhibits target chromatins through the trimethylation of Histone 3 lysine 27 (H3K27me3) (Cao et al., 2002; Czermin et al., 2002; Müller et al., 2002; Nekrasov et al., 2005). PRC2 controls FM initiation, organ identity specification, and meristem termination (Gan et al., 2013).

The PRC2 has several components. In *Arabidopsis*, mutants of the core components of PRC2 – CLF/SWN, FIE, EMF2, and MSI – present ectopic expression of *AGAMOUS* (*AG*), causing abnormal floral phenotypes similar to those of *AG*-overexpression plants (Goodrich et al., 1997; Yoshida et al., 2001; Hennig et al., 2003; Moon et al., 2003; Katz et al., 2004). This complex also influences FM termination by regulating the temporal expression of *WUSCHEL* (*WUS*) and *KNUCKLES* (*KNU*) (Mozgova et al., 2015). After all of the floral organs are initiated, FM is terminated through the repression of *WUS*, a meristem maintenance gene (Mayer et al., 1998). PRC2 inhibits the expression of *KNU*, which inhibits *WUS* transcription (Lenhard et al., 2001; Sun et al., 2009). For floral termination, activated *AG* displaces PRC2 from *KNU*, leading to activation of *KNU* and repression of *WUS* (Sun et al., 2014).

VERNALIZATION INSENSITIVE 3 (VIN3), another component of PRC2, enhances H3K27me3 in *FLOWERING LOCUS C* (*FLC*), a repressor of flowering (Sung and Amasino, 2004). In rice, the VIN3-LIKE protein OsVIL2 enhances flowering by mediating H3K27me3 on chromatin of *LFL1*, which is a negative regulator of flowering (Yang et al., 2013). OsVIL2 binds to OsEMF2b, an ortholog of *Arabidopsis* EMF2 (Yang et al., 2013). Mutants in *OsEMF2b* display phenotypes of severe floral organ defects and meristem indeterminacy, similar to the mutants defective in E-function genes *OsMADS1, OsMADS6*, and *OsMADS34* (Luo et al., 2009; Yang et al., 2013; Conrad et al., 2014; Xie et al., 2015). OsEMF2b represses the expression of these E-function genes by altering H3K27me3 on their chromatins (Conrad et al., 2014). In addition, OsEMF2b inhibits *OsLFL1* and *OsMADS4* by mediating H3K27me3 on their chromatin, resulting in the promotion of flowering and regulating the specification of floral organ identity (Xie et al., 2015).

The *osvil2* mutants also display abnormal spikelet development. In this study, we studied the mutant phenotypes that appear in the early stages of that process and performed transcriptome analysis to identify downstream genes controlled by *OsVIL2*.

## Materials and Methods

### Plant Materials and Growth Conditions

Two *OsVIL2* mutant lines, *osvil2-1* and *osvil2-2*, were isolated from a pool of rice T-DNA tagging lines (Jeon et al., 2000b; Jeong et al., 2002; Yang et al., 2013). Seeds of the mutants and wild type (WT) were germinated on MS media and genotyping was conducted to identify homozygous plants, as previously explained (Yi and An, 2013). Plants were grown either in the paddy field under natural conditions or in a greenhouse under supplemental, artificial lighting.

### Vector Construction and Rice Transformation

For *OsVIL2* promoter – *GUS* fusion construction, we used the 2,348-bp promoter fragment between -2340 and +8 from the translation initiation site of *OsVIL2* that was used for complementation of the mutant (Zhao et al., 2010). The fragment was amplified by PCR and placed upstream of the promoterless *GUS* gene using the pGA3519 binary vector that contains hygromycin selectable marker. The primer sequences for amplification of the promoter region are listed in **Supplementary Table S1**. *Agrobacterium tumefaciens* strain LBA4404 was transformed with this vector by the freeze-thaw method (An et al., 1989). Transgenic plants expressing the *GUS* reporter gene were generated by co-cultivating the *Agrobacterium* cells with scutellum calli derived from mature seeds of rice (cv. Longjin). The co-cultivated calli were selected and regenerated as previously reported (Jeon et al., 1999).

### Histochemical GUS Staining

Plant tissues were submerged in GUS-staining solution that contained 100 mM sodium phosphate (pH 7.0), 0.1 mM potassium ferricyanide, 0.1 mM potassium ferrocyanide, 0.1% Triton X-100, 10 mM EDTA (pH 8.0), 1% DMSO, 0.1% X-gluc (5-bromo-4-chloro-3-indolyl-β-D-glucuronic acid/cyclohexylammonium salt), and 5% methanol (Yoon et al., 2014). Samples were incubated overnight at 37°C, then transferred to 70% ethanol at 65°C for several hours to remove chlorophylls before being stored in 95% ethanol.

### Scanning Electron Microscopy

Specimens were prepared as previously described (Lee et al., 2007). Samples were fixed in FAA solution, dehydrated in an ethanol series, and critical point-dried in a Leica EM CPD300 (Leica Microsystems, Wetzlar, Germany). They were mounted on stubs, sputter-coated with platinum, and observed under a scanning electron microscope (SIGMA FE-SEM; Carl Zeiss, Germany).

### RNA Isolation and qRT-PCR Analysis

Total RNAs were isolated with RNAiso Plus (TaKaRa, Shiga, Japan). The cDNAs were synthesized with 2 μg of RNA, Moloney murine leukemia virus reverse transcriptase (Promega, Madison, WI, United States), RNasin Ribonuclease Inhibitor (Promega), 10 ng of the oligo (dT)_18_ primer, and 2.5 mM deoxyribonucleotide triphosphates. Quantitative RT-PCR was conducted with SYBR Green I Prime Q-Mastermix (GENETBIO, Daejeon, South Korea) and a Rotor-Gene 6000 (Corbett Research, Sydney, NSW, Australia), following protocols reported earlier (Yang et al., 2013; Wei et al., 2016). Rice *Ubiquitin1* was used as an internal control for quantitative real-time PCR (qRT-PCR).

For transcriptome analyses, total RNAs were prepared from 2- and 4-mm panicles of WT and *osvil2-1* plants, in three biological replicates. Double-stranded cDNAs were synthesized with random hexamers and ligated to adaptors. This library was pair-end sequenced using the PE90 strategy on an Illumina HiSeq^TM^ 2000 at the Beijing Genomics Institute (Wuhan, China). The DEseq algorithm was applied to filter the differentially expressed genes. All Gene Ontology (GO) annotations were downloaded from NCBI^1^, UniProt^2^, and the Gene Ontology website^3^. The RNA sequencing data were deposited to the GEO database (accession number: GSE108538).

## Results

### Mutations in *OsVIL2* Caused Abnormal Spikelet Formation

**FIGURE 1:**
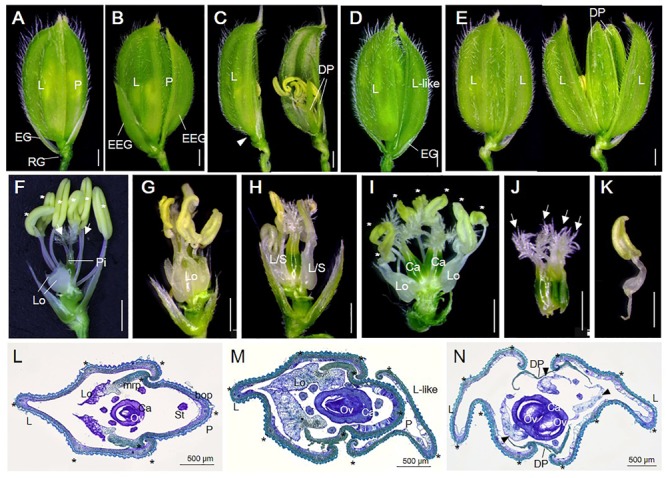
Spikelet phenotypes of WT and *osvil*2 mutant. **(A–E)** Phenotypes of WT and *osvil2-1* spikelets. **(A)** WT spikelet with pair of rudimentary glumes and empty glumes, lemma, palea. **(B)** *osvil2-1* spikelet with elongated empty glumes. **(C)** *osvil2-1* spikelet with degenerated palea and no empty glume on lemma side (arrowhead). **(D)** *osvil2-1* spikelet with additional lemma-like organ. **(E)** *osvil2-1* spikelet with 2 lemma and 2 degenerated palea. **(F–K)** Phenotypes of inner floral organs of WT and *osvil2-1*. Palea and lemma were removed. **(F)** WT spikelet comprises two lodicules on lemma side, six stamens (asterisks), and one pistil with two stigmas (arrows). **(G)** *osvil2-1* floret with extra lodicules and immature stamens. **(H)** *osvil2-1* floret with lodicule–stamen mosaic organs. **(I)** *osvil2-1* floret with extra lodicules, seven stamens (asterisks), and two carpels. **(J)** *osvil2-1* carpel in which several carpels are fused. Number of stigma is also increased (arrows). **(K)** Lodicule–stamen mosaic organ in *osvil2-1*. **(L)** Cross section of WT spikelet. **(M,N)** Cross section of *osvil2* spikelet. **(M)** Formation of additional lemma-like organ in *osvil2* spikelet. **(N)** *osvil2* with two lemma, two degenerated palea, three abnormal lodicules (arrow heads), and one fused carpel having two ovules. bop, body of palea; Ca, carpel; DP, depressed palea; EG, empty glume; EEG, elongated empty glume; L, lemma; Lo, lodicule; L/S, lodicule–stamen mosaic organ; L-like; lemma-like organ; mrp, marginal region of palea; Ov, ovule; P, palea; RG, rudimentary glume. Asterisks indicate stamens **(F,I)** or vascular bundles **(L–N)**. Scale bars = 1 mm **(A–K)** or 500 μm **(L–N)**.

**FIGURE 2:**
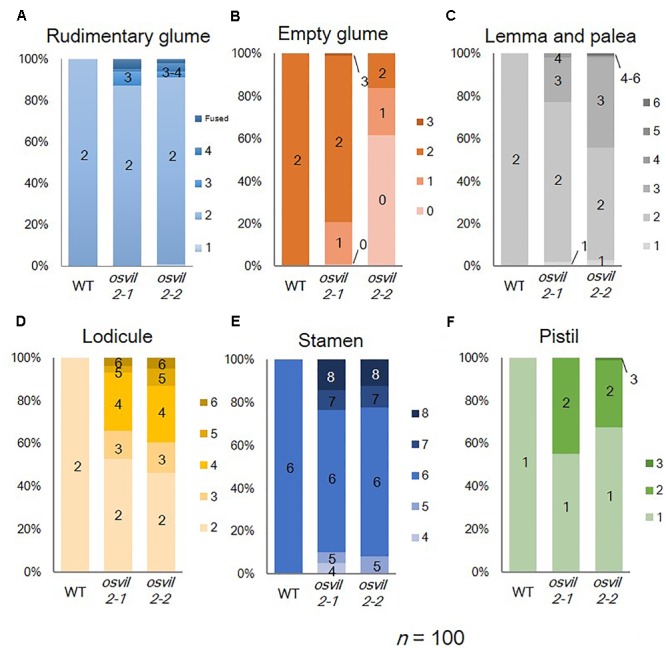
Floral organ numbers in WT, *osvil2-1*, and *osvil2-2* spikelets. **(A)** Rudimentary glumes; **(B)** empty glumes; **(C)** lemma and palea; **(D)** lodicules; **(E)** stamens; **(F)** pistils.

We previously showed that T-DNA insertional mutant lines *osvil2-1* and *osvil2-2* display pleiotropic phenotypes, including late-flowering, fewer tillers, and abnormal spikelet development (Yang et al., 2013). In this study we characterized the spikelet defects in detail (**Figure 1** and **Supplementary Figure S1**). The WT spikelets had a pair of rudimentary glumes plus empty glumes (**Figure 1A**). In contrast, the number of rudimentary glumes was increased in 14% of the *osvil2* spikelets (**Figure 2A**). Furthermore, the empty glumes were elongated in 21% of the mutant spikelets (**Figure 1B** and **Supplementary Figure S1B**), while the number of empty glumes was decreased in 21%, resulting in spikelets with one or no empty glumes (**Figures 1C, 2B**). Occasionally, a lemma-like organ developed at the position of the empty glume. These observations indicated that mutations of *OsVIL2* affected both the number and morphology of empty glumes.

In addition, the development of all floral organs was abnormal in the *osvil2* florets. Extra lemma-like structures were observed in 45% of the *osvil2* spikelets, creating three or more such structures (**Figures 1D, 2C** and **Supplementary Figure S1C**). Additional lemma-like organs were often produced at an ectopic whorl. Some of those ectopic lemma-like organs resembled lemma (**Figure 1D**). The number of lemma was increased in 14% of the spikelets from *osvil2*, and they often accompanied degenerated palea (**Figures 1E, 2C**). The development of palea was defective in 33% of those mutants (**Figure 1C** and **Supplementary Figure S1D**), and cross sections of the spikelets showed extra lemma-like organs (**Figure 1M**) and degenerated palea (**Figure 1N**).

The number of lodicules increased to three or more in 54% of the *osvil2* spikelets (**Figures 1G–I, 2D** and **Supplementary Figures S1F,G**), and their morphology was occasionally abnormal. Lodicules were elongated (20%) or an anther-like organ formed in the upper part of 26% of those lodicules (**Figures 1 H,I,K**). The number of stamens decreased in 8% of the spikelets (**Figures 1H, 2E**) but increased in 22% (**Figures 1I, 2E**). Finally, the number of carpels rose to two in 32% of the mutant spikelets (**Figures 1I, 2F**) and they were often fused (**Figures 1 H,J**). These observations indicated that *OsVIL2* is needed for proper development of all organs within a spikelet.

### Early Spikelet Development Was Affected in *osvil2*

**FIGURE 3:**
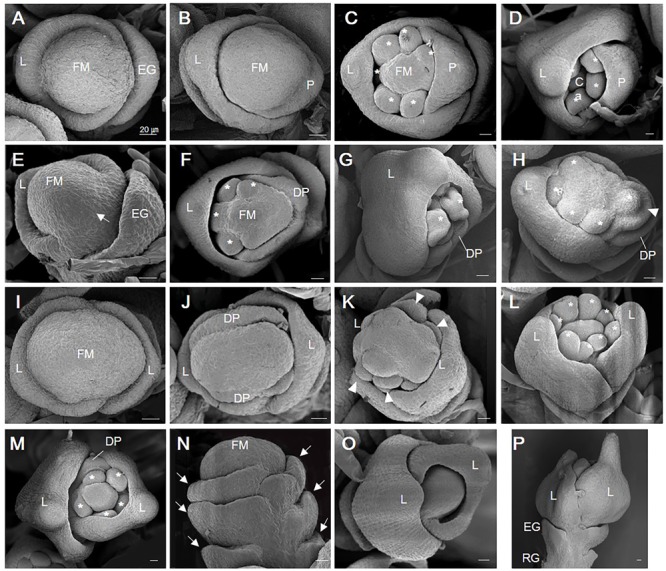
Scanning electron micrographic images of WT and *osvil2* spikelets during early developmental stages. **(A–D)** WT spikelets. **(A)** Stage Sp3 during lemma primordia formation. **(B)** Stage Sp4-5 of palea primordia formation; possibly two lodicule primordia are initiating inside lemma. **(C)** Stage Sp6, with formation of six stamen primordia (asterisk). **(D)** Stage Sp8, with carpel primordia initiation during growth of lemma and palea. **(E–H)** Retarded palea growth in *osvil2* spikelets. **(E)** Stage Sp4, showing retardation of palea primordia (arrow) when compared with lemma growth. **(F)** Stage Sp6, with degenerated palea formation and retarded stamen primordia formation on palea side. **(G)** Stage Sp8, with retarded growth of palea compared with lemma. **(H)** Stage Sp6, showing formation of stamen primordia (asterisk) in irregular pattern. Additional organ primordia (arrow head) between palea and stamen, increasing size of floral whorl. **(I–L)** *osvil2* spikelet with twin-flower phenotype. **(I)** Stage Sp3, with wider floral meristem and two lemma primordia on both sides. **(J)** Stage Sp4, with two degenerated palea primordia forming in middle of two lemma. **(K)** Stage Sp6, representing ectopic organ primordia (arrow heads) likely to produce additional palea and lodicules. **(L)** Stage Sp6-7, with eight stamen primordia produced. **(M–P)** *osvil2* spikelet with additional glume or lemma-like organs. **(M)** Stage Sp7, in which number of stamen primordia is reduced to 5. Floral whorls are increased and degenerated palea form after additional lemma. **(N)** Stage Sp4, with formation of additional glume primordia (arrows). **(O)** Stage Sp6, in which additional lemma are produced while inner organ formation is delayed. **(P)** Stage Sp8, with additional lemma produced after formation of normal empty glumes and rudimentary glumes. Ca, carpel; DP, degenerated palea; EG, empty glume; FM, floral meristem; L, lemma; P, palea; RG, rudimentary glume. Scale bars = 20 μm.

We used a scanning electron microscope to study spikelet defects during early developmental stages that were classified based on the previous research (Ikeda et al., 2004). In the WT, the FM produced an empty glume and a lemma on the opposite side of the empty glume during spikelet developmental stage Sp3 in the WT (**Figure 3A**). Palea subsequently developed in Stages 4–5 (**Figure 3B**). While the lemma and palea were elongating, stamens developed at Stage 6 (**Figure 3C**). Finally, a carpel arose at the central position of the spikelet at Stage 8 (**Figure 3D**).

In *osvil2* spikelets, the formation of palea primordia was often retarded at Sp4 (**Figure 3E**). At Sp6, the mutant spikelets displayed degenerated palea primordia, often along with retarded development of inner floral organs on the palea side (**Figures 3F,G**). This retardation seemed to cause a decline in the number of stamens produced as well as abnormal development of inner floral organs (**Figure 3H**). The FM were larger in some *osvil2* spikelets that usually accompanied two lemma primordia and two degenerated palea primordia (**Figures 3I,J**). At Sp6, additional lodicule primordia were observed between the lemma and stamen primordia (**Figure 3K**). During that stage, the number of stamen primordia was altered (**Figures 3L,M**), and reiterative formation of glumes occurred occasionally (**Figure 3N**). Extra glumes and lemma-like organs were produced at additional whorls (**Figures 3M–P**). These findings indicated that *OsVIL2* functions during the early stages of spikelet development.

### Expression Pattern of *OsVIL2*

**FIGURE 4:**
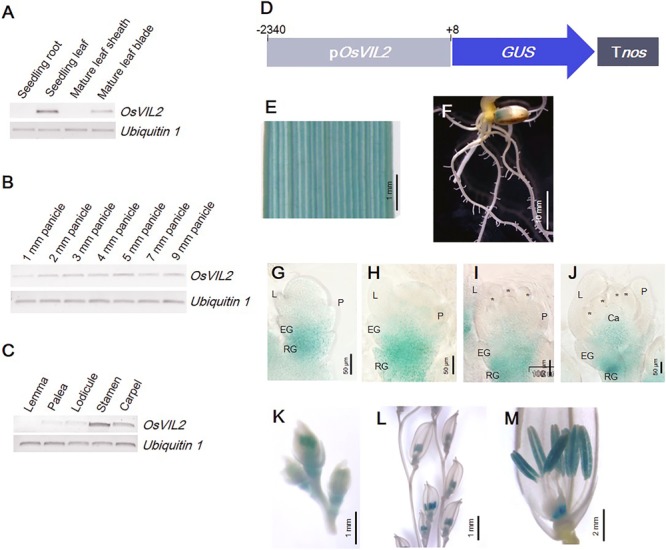
Expression pattern of *OsVIL2* in various organs. **(A–C)** RT-PCR expression analysis. **(A)** Expression in various organs. **(B)** Expression during early spikelet development. **(C)** Expression in each floral organ within mature spikelets. **(D)** Schematic diagram of *pOsVIL2*::*GUS* construct. **(E–M)** Observation of promoter-GUS trapping line of *OsVIL2.* Expression in leaf blade **(E)**, root **(F)**, spikelet at Sp4 **(G)**, spikelet at Sp5 **(H)**, spikelet at Sp6 **(I)**, spikelet at Sp7 **(J)**, spikelets in 12-mm young panicle **(K)**, spikelets in 50-mm panicle **(L)**, and mature spikelet **(M)**. EG, empty glume; L, lemma; P, palea; RG, rudimentary glume.

The pattern of *OsVIL2* expression was analyzed by RT-PCR. In vegetative tissues, the gene was detected in seedling leaves and in the leaf blades of mature plants (**Figure 4A**). It was also constitutively expressed in panicles at various developmental stages (**Figure 4B**). In mature spikelets, expression was strong in the stamens and carpels and weak in the lodicules and palea (**Figure 4C**). Expression was also studied at the tissue level by using the promoter region of *OsVIL2* fused to the *GUS* reporter gene (**Figure 4D**). We obtained 13 plants independently transformed with this *OsVIL2* promoter-*GUS* construct. Those lines displayed similar expression pattern, thus we selected the line with the highest GUS expression for further analysis. The *GUS* reporter was expressed strongly in leaves but not expressed in roots, as observed from the RT-PCR analyses (**Figures 4E,F**). During spikelet development, the reporter was detected in the basal regions of the spikelets (**Figures 4G–J**). In florets at Sp8, it was strongly expressed in anthers and the basal region of carpels (**Figures 4K–M**). This organ-preferential expression pattern was consistent with the pattern revealed from the qRT-PCR data.

### Transcriptome Analyses of Young Panicles

**FIGURE 5:**
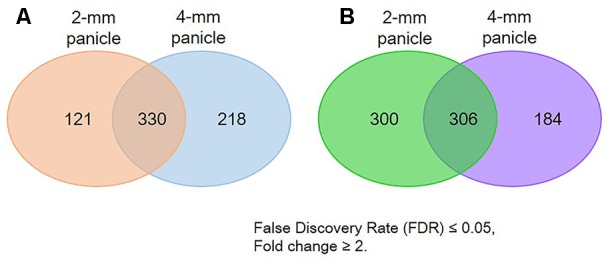
Number of genes differentially expressed in *osvil2* young panicles. **(A)** Number up-regulated; **(B)** number down-regulated.

RNA-sequencing assays were conducted to identify genes differentially expressed in *osvil2* during early spikelet development. Two stages were examined: 2-mm panicles containing spikelets mostly at Sp4 or earlier, and 4-mm panicles containing spikelets at mainly Sp7 or younger. In the 2-mm samples, 451 genes were up-regulated (**Supplementary Table S2**) and 606 genes were down-regulated (**Supplementary Table S3**) by at least twofold. In the 4-mm samples, 548 genes were up-regulated (**Supplementary Table S4**) and 490 genes were down-regulated (**Supplementary Table S5**) by at least twofold. The overlap between 2- and 4-mm samples showed that 330 genes were up-regulated (**Supplementary Table S6**) while 306 were down-regulated (**Supplementary Table S7**). In total, 669 genes were up-regulated and 790 genes were down-regulated by at least twofold in both size classes (**Figure 5**).

We performed GO analysis using the differentially expressed genes (**Supplementary Figure S2**). Our GO analysis revealed that Flower Development (GO:0009908) was significantly enriched for the downregulated genes in 2-mm panicles, which implied that the regulatory genes involved in that process were suppressed in *osvil2* at the early stages. Terms for Transcription (GO:0006351) and Regulation of Transcription (GO:0006355) were also highly enriched for the downregulated genes from both panicle sizes (**Supplementary Figures S2B,D**). Among the transcription factors, many of the AP2 and MADS-box family genes were significantly down-regulated (**Supplementary Table S3**). Because several genes within those families play important functions in SM formation and floral organ development, their downregulation in developing panicles was likely the reason for the abnormal spikelet phenotypes.

Transcriptome analyses of the genes involved in meristem phase transition or spikelet organ development are shown in **Table 1**. Genes determining meristem size – *FON1, FON4*, and *OsWUS* (Suzaki et al., 2004; Chu et al., 2006; Moon et al., 2006; Nardmann and Werr, 2006) – were almost equally expressed in the *osvil2* mutants. Among the genes that function in the transition from inflorescence meristem (IM) to SM, the transcriptome frequency of *APO1* was significantly reduced in both panicle sizes. *APO1*, an ortholog of *Arabidopsis UFO*, suppresses earlier conversion of IM to SM and promotes cell proliferation in the IM (Ikeda et al., 2005, 2007; Ikeda-Kawakatsu et al., 2009). In addition, *APO1* regulates floral organ identity and floral determinacy by enhancing expression of the C-function gene *OsMADS3* (Ikeda et al., 2005). However, we found that the level of *APO2*/*RFL* expression was increased in our *osvil2* mutants. The *APO2*/*RFL* gene, an ortholog of *Arabidopsis LFY*, also suppresses the transition from IM to SM. A reduction in its expression decreases the number of panicle branches produced and alters floral organ identity (Rao et al., 2008; Ikeda-Kawakatsu et al., 2012).

**Table 1:** Expression levels for genes that control spikelet development.

Function	Gene	Panicle 2 mm			Panicle 4 mm		
		WT	*osvil2*	FC	WT	*osvil2*	FC
Meristem size (meristem maintenance)	*FON1*	771.4	615.0	0.80	486.8	497.0	1.02
	*FON2/FON4*	86.5	54.3	0.63	49.1	40.3	0.82
	*OsWUS*	24.3	19.2	0.79	23.0	14.1	0.61
Transition from IM to SM/FM	*TAW*	135.6	107.5	0.79	110.0	74.2	0.67
	*APO1*↓	57.4	20.9	0.36	28.0	9.7	0.35
	*APO2/RFL*↑	3280.0	4908.4	1.50	2296.3	3617.8	1.58
	*RCN1*	16.5	20.2	1.22	15.8	16.8	1.06
	*RCN2*	17.5	6.4	0.37	1.6	7.7	4.68
	*RCN3*	110.3	142.1	1.29	131.4	127.8	0.97
	*RCN4*↑	22.4	54.5	2.44	26.0	58.8	2.26
Transition SM to FM	*FZP*↑	65.6	106.6	1.63	47.5	66.5	1.40
	*SNB*	1230.8	1228.1	1.00	1248.3	1245.4	1.00
	*MFS*	1174.3	1234.0	1.05	925.8	932.1	1.01
SM regulation, empty glume development	*TOB1*	3258.4	2436.3	0.75	3866.1	2888.7	0.75
	*EG1*	35.5	42.8	1.21	29.0	32.8	1.13
	*OsJAZ1/EG2*	4448.5	3617.2	0.81	4206.7	3676.2	0.87
	*OsIG1*↓	445.2	188.7	0.42	556.9	312.2	0.56
	*G1/ELE*↓	553.6	103.6	0.19	610.4	103.9	0.17
Palea development	*REP1*↓	44.2	23.6	0.53	48.1	20.8	0.43
	*DP1*↓	347.6	269.2	0.77	248.7	142.2	0.57
Floral organ identity	*OsMADS32*	4715.4	5236.4	1.1	3968.8	4403.9	1.1
A Function	*OsMADS14*	7971.6	8450.6	1.06	7272.7	7936.4	1.09
	*OsMADS15*	8425.3	10605.0	1.26	8519.2	9816.5	1.15
	*OsMADS18*	4468.5	4804.5	1.08	4481.5	4900.4	1.09
B Function	*OsMADS2*	1656.4	1140.9	0.69	1684.0	1481.1	0.88
	*OsMADS4*↓	401.0	119.2	0.30	552.7	289.5	0.52
	*OsMADS16*↓	1377.4	434.0	0.32	1709.9	1144.7	0.67
C Function	*OsMADS3*↓	635.2	194.3	0.31	518.4	298.3	0.58
	*OsMADS58*↓	247.9	61.0	0.25	258.9	130.1	0.50
	*DL*↓	1033.4	616.3	0.60	1059.8	947.3	0.89
D Function	*OsMADS13*↓	62.2	5.0	0.08	60.0	26.5	0.44
E Function	*OsMADS1*↓	4493.6	2096.6	0.47	4592.3	2667.2	0.58
	*OsMADS5*↓	2328.2	3562.7	1.53	3562.7	2328.2	1.53
	*OsMADS7*↓	2374.8	884.2	0.37	2975.9	1963.5	0.66
	*OsMADS8*↓	2361.2	629.6	0.27	2022.9	1135.6	0.56
	*OsMADS34*↓	2908.0	3619.6	1.24	2709.0	3666.4	1.35
	*OsMADS6*↓	5235.3	1839.8	0.35	3874.1	2070.0	0.53

Among the four *RCN* genes that are homologous to *Arabidopsis TERMINAL FLOWER 1* (Nakagawa et al., 2002), expression of *RCN4* was increased by at least twofold in the mutant spikelets, while that of *RCN1, RCN2*, and *RCN3* was not significantly affected. Furthermore, we were unable to find any significant change in transcriptome levels for *SNB* and *MFS*, which function during the transition from SM to FM (**Table 1**). Expression was slightly elevated for *FZP*, a gene that inhibits the axillary meristem in spikelets and promotes FM by enhancing the expression of B-function (*OsMADS4* and *OsMADS16*), E-function (*OsMADS1, OsMADS7*, and *OsMADS8*), and AGL6-like (*OsMADS6* and *OsMADS17*) MADS-box genes (Komatsu et al., 2003; Bai et al., 2016).

Among the genes that are necessary for the formation of empty glumes (Yoshida et al., 2009; Zhang et al., 2015), *G1* and *OsIG1* were significantly down-regulated in young spikelets from *osvil2*. Because empty glume development was abnormal and their numbers were fewer in the mutant, reduced gene expression may have been responsible for these defects. Moreover, downregulation of *REP1*, which is requires for palea development (Yuan et al., 2009), may have been related to the degeneration of palea in *osvil2*.

Several MADS-box genes that function in floral organ identity were down-regulated in the *osvil2* spikelets. Expression of the B-function *OsMADS4* and *OsMADS16* was affected in the mutant, especially in 2-mm spikelets. The C-function *OsMADS3, OsMADS58*, and *DL* were also down-regulated in the mutant spikelets. In addition, expression was significantly reduced for the D-function *OsMADS13* and the E-function *OsMADS1, OsMADS6, OsMADS7*, and *OsMADS8*. In contrast, expression of A-function genes *OsMADS14, OsMADS15*, and *OsMADS18* was not significantly affected while that of *OsMADS5* was increased.

**FIGURE 6:**
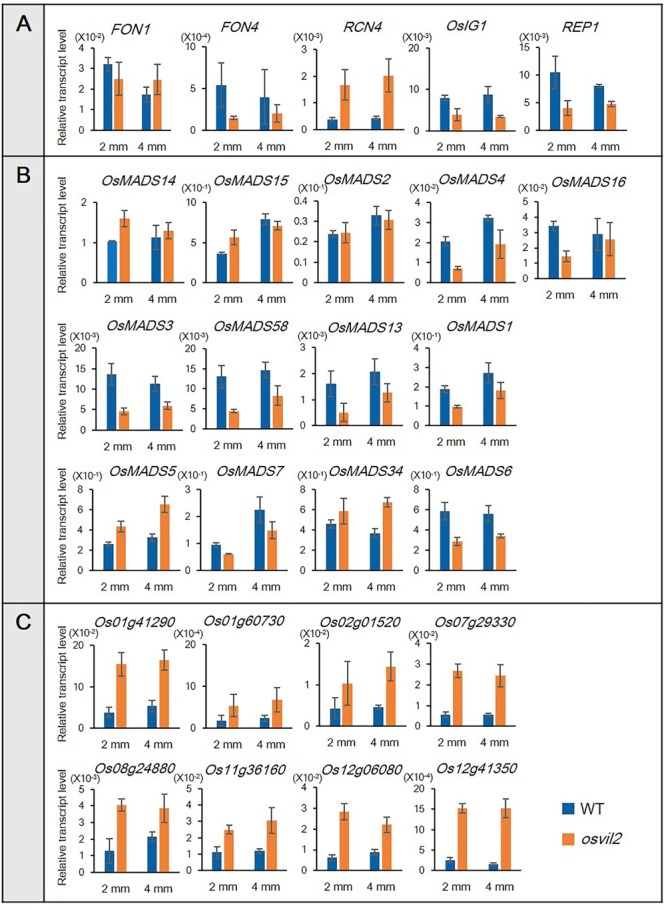
Verification of RNA-sequencing results via qRT-PCR. Transcript levels are relative to *Ubi1*. **(A)** qRT-PCR analysis of genes involved in spikelet development. **(B)** qRT-PCR analysis of MADS-box genes involved in floral organ development. **(C)** qRT-PCR analysis of upregulated genes previously identified in RNA-sequencing analysis.

To verify our RNA-sequencing data, we conducted qRT-PCR, focusing on 13 MADS-box genes that control floral organ identity and development (**Figure 6**). Among them, eight (*OsMADS1, OsMADS3, OsMADS4, OsMADS6, OsMADS7, OsMADS13, OsMADS16*, and *OsMADS58*) were down-regulated while the expression of five (*OsMADS2, OsMADS5, OsMADS14, OsMADS15*, and *OsMADS34*) was not changed significantly, according to the transcriptome analyses (**Figure 6B**). In the *osvil2* mutants, expression of all eight downregulated genes was significantly reduced, based on the qRT-PCR results. Among the five for which RNA-sequencing analyses showed no significant change, expression was similar between WT and *osvil2* for three, while two others, *OsMADS5* and *OsMADS34*, were slightly up-regulated in the mutant panicles.

We also selected eight genes shown to be up-regulated based on our transcriptome analyses (**Figure 6C**). The qRT-PCR verification experiments revealed that their expression was much higher in the *osvil2* mutants. All of these outcomes demonstrated that the data obtained from RNA-sequencing analyses are reliable.

## Discussion

In this study, we analyzed the abnormal phenotypes of *osvil2* and found that they were variable in all organs of the spikelets. Furthermore, expression of essential regulatory genes for spikelet development was significantly altered in the mutant. These results demonstrated how necessary *OsVIL2* is for normal organ patterning during spikelet formation because it modulates proper expression of those genes that control organ development and identity in spikelets.

The *osvil2* mutant spikelets produced elongated empty glumes that resembled lemma. Homeotic transformation of empty glumes into lemma has also been described for mutants defective in *G1, EG1, EG2, OsMADS34*, and *OsIG1* (Li et al., 2009; Yoshida et al., 2009; Gao et al., 2010; Cai et al., 2014; Zhang et al., 2015). As the empty glumes are not found in the spikelets of other grasses, the origin and identity of empty glume are controversial (Yoshida and Nagato, 2011). Elongated empty glume phenotype of *osvil2* supports the idea that the empty glumes are degenerated lemma of sterile florets (Arber, 1934). Expression of genes for empty glume identity – *G1* and *OsIG1* – was substantially lower in the developing panicles of *osvil2* than in those of the WT, suggesting that *OsVIL2* functions upstream of *G1* and *OsIG1* to specify sterile lemma identity.

Another phenotype of *osvil2* spikelets was the formation of degenerated palea. This defect occurred mostly in the palea body rather than in the marginal region. In addition, the middle portion of the palea primordia often did not grow, thereby splitting the palea. These phenotypes are similar to those reported for mutants defective in *REP1, OsMADS15, MFS1, DP1*, or *OsIG1* (Yuan et al., 2009; Wang et al., 2010; Jin et al., 2011; Ren et al., 2013; Zhang et al., 2015). Expression of *REP1, DP1*, and *OsIG1* was reduced in *osvil2* mutants, suggesting that those genes are linked with the palea defects. In *Arabidopsis, VIL* genes function in vernalization and flowering time (Sung and Amasino, 2004; Sung et al., 2006; Greb et al., 2007). Our observation that rice *VIL* functions in spikelet development indicates a diversified function of the gene family. Because spikelet is a unique structure of grass inflorescence, it will be interesting to study whether function of *OsVIL2* is conserved in other grass species.

The most significant phenotype of *osvil2* was an increase in numbers for all floral organs. This phenotype was similar to that of mutants defective in *FON4*, an ortholog of *Arabidopsis CLV3* (Chu et al., 2006). There, the number of floral organs in the inner whorls is more highly affected in the spikelets. For *fon4* mutants, the carpel number can increase up to 10 whereas that number rose to two in *osvil2*. Stamen numbers also increase up to 10 in *fon4* versus up to eight in *osvil2*. The homeotic conversion of empty glumes to lemma is common to both *fon4* and *osvil2* mutants. We noted that expression of *FON4* was reduced in the developing *osvil2* panicles, suggesting that this gene functions downstream of *OsVIL2*.

Floral organ identity is regulated by numerous genes, including some MADS-box genes (Zhang and Yuan, 2014). We observed that several MADS-box genes were differentially expressed in *osvil2*. Alteration in the expression of these floral homeotic genes may have been responsible for the abnormal development of floral organs in the mutant.

This study revealed that *OsVIL2* affects variable aspects of spikelet development by controlling various genes important for spikelet development. Because OsVIL2 functions together with PRC2, which suppresses target chromatin, we expected to find that expression of direct targets would be higher in the *osvil2* mutants. Instead, transcription levels for most regulatory genes that control organ number or identity were reduced, while expression was slightly increased for *RCN4, OsMADS5*, and *OsMADS34*. This implied that they may be direct targets of OsVIL2-PRC2. Although the function of *RCN4* remains unknown, overexpression of *RCN1* and *RCN2* can result in highly branched panicles, suggesting that they also have roles in suppressing floral fate (Nakagawa et al., 2002). In an earlier functional study of *OsEMF2b, OsMADS34* was predicted as a direct target gene of *Os*EMF2b (Conrad et al., 2014). Because OsEMF2b interacts with OsVIL2 (Yang et al., 2013), mutations of *OsVIL2* may also influence the MADS-box genes.

## Author Contributions

GA organized the entire of this research. GA, HY, JY, WL, and DZ designed the research. HY, JY, WL, and DZ performed the experiments and analyzed data. HY and GA wrote the manuscript. All authors read and approved the manuscript.

## Conflict of Interest Statement

The authors declare that the research was conducted in the absence of any commercial or financial relationships that could be construed as a potential conflict of interest.

## Footnotes

1 http://www.ncbi.nlm.nih.gov/

2 http://www.uniprot.org/

3 http://www.geneontology.org/

## Supplementary Material

FIGURE S1 Phenotypes of *osvil2-2* spikelets. **(A)** Abnormal rudimentary glume formation in *osvil2-2* spikelet. **(B)** *osvil2-2* spikelet with elongated empty glumes. **(C)** *osvil2-2* spikelet with additional lemma-like organs, indicated by arrows. **(D)** *osvil2-2* spikelet with degenerated palea. **(E)** Twin-flower phenotype. **(F)** Additional floral organ formation. **(G)** Additional lodicule and elongated lodicule formation. **(H)** Abnormal carpel with increased number of stigmas and undifferentiated cell mass. **(I)** Lodicule–stamen mosaic organ in *osvil2-2*. Ca, carpel; EEG, elongated empty glume; Elo, elongated lodicule; L, lemma; Lo, lodicule; P, palea; ucm, undifferentiated cell mass. Scale bars = 1 mm.

FIGURE S2 Geneontology analysis of differentially expressed genes in *osvil2*. **(A)** Enrichment of upregulated genes in 2-mm panicle. **(B)** Enrichment of downregulated genes in 2-mm panicle. **(C)** Enrichment of upregulated genes in 4-mm panicle. **(D)** Enrichment of downregulated genes in 4-mm panicle.

Table S1 Sequences of primers used in this study.

TABLE S2 Genes up-regulated in 2-mm young panicles of *osvil2*.

TABLE S3 Genes down-regulated in 2-mm young panicles of *osvil2*.

TABLE S4 Genes up-regulated in 4-mm young panicles of *osvil2*.

TABLE S5 Genes down-regulated in 4-mm young panicles of *osvil2*.

TABLE S6 Genes up-regulated in both 2- and 4-mm young panicles of *osvil2*.

TABLE S7 Genes down-regulated in both 2- and 4-mm young panicles of *osvil2*.
