# Supplementary figures and images for: OsVIL2 Regulates Spikelet Development by Controlling Regulatory Genes in Oryza sativa
Source: Front Plant Sci. 2018 Feb 6;9:102. doi: 10.3389/fpls.2018.00102 (PMC5808121; doi:10.3389/fpls.2018.00102)

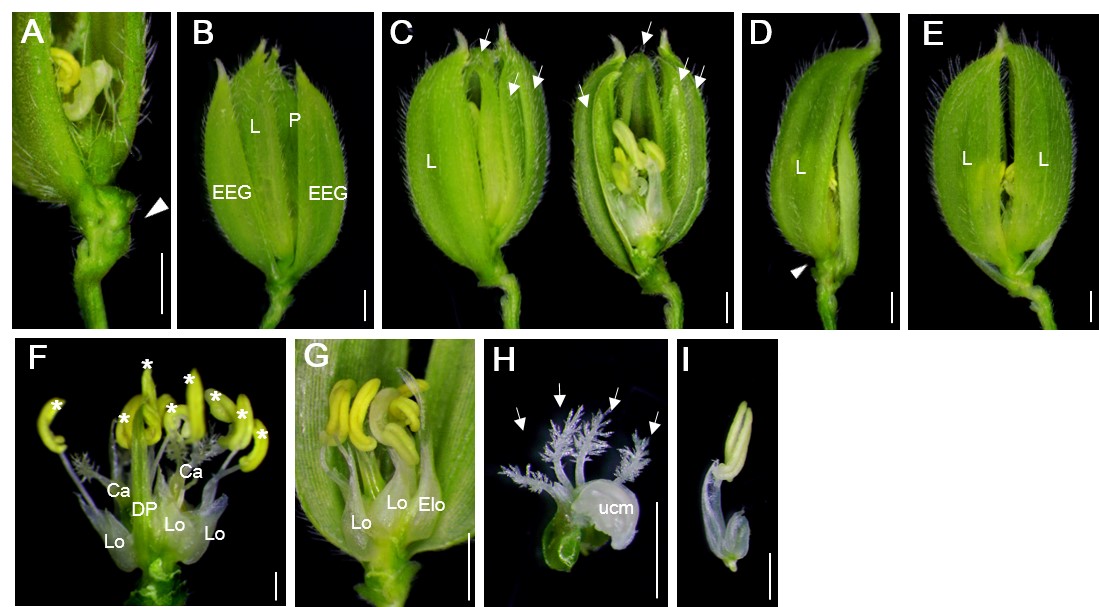

Supplement: FIGURE S1 — Phenotypes of osvil2-2 spikelets. (A) Abnormal rudimentary glume formation in osvil2-2 spikelet. (B) osvil2-2 spikelet with elongated empty glumes. (C) osvil2-2 spikelet with additional lemma-like organs, indicated by arrows. (D) osvil2-2 spikelet with degenerated palea. (E) Twin-flower phenotype. (F) Additional floral organ formation. (G) Additional lodicule and elongated lodicule formation. (H) Abnormal carpel with increased number of stigmas and undifferentiated cell mass. (I) Lodicule–stamen mosaic organ in osvil2-2. Ca, carpel; EEG, elongated empty glume; Elo, elongated lodicule; L, lemma; Lo, lodicule; P, palea; ucm, undifferentiated cell mass. Scale bars = 1 mm. [file Image_1.JPEG]

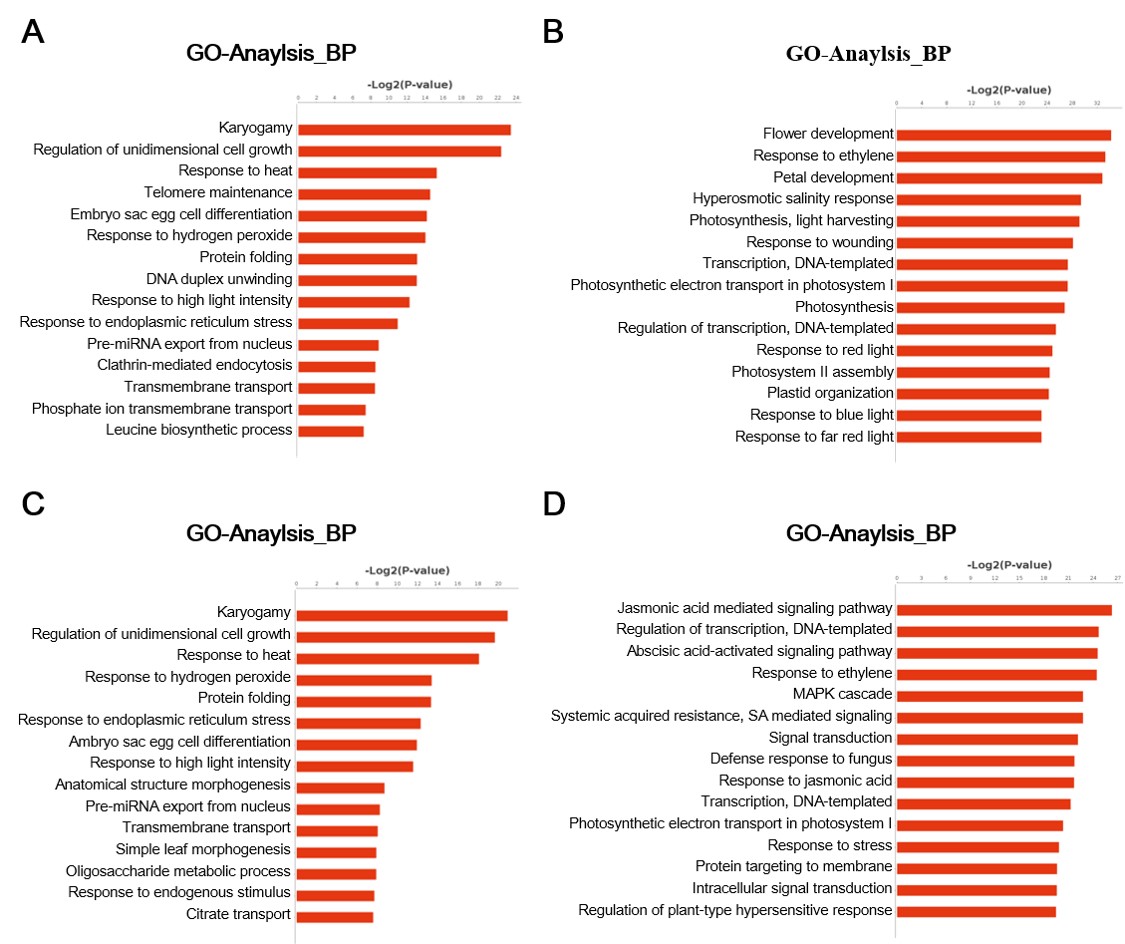

Supplement: FIGURE S2 — Geneontology analysis of differentially expressed genes in osvil2. (A) Enrichment of upregulated genes in 2-mm panicle. (B) Enrichment of downregulated genes in 2-mm panicle. (C) Enrichment of upregulated genes in 4-mm panicle. (D) Enrichment of downregulated genes in 4-mm panicle. [file Image_2.JPEG]
