# Supplementary material for: OsVIL2 Regulates Spikelet Development by Controlling Regulatory Genes in Oryza sativa
Source: Front Plant Sci. 2018 Feb 6;9:102. doi: 10.3389/fpls.2018.00102 (PMC5808121; doi:10.3389/fpls.2018.00102)
Supplement: Table S1 — Sequences of primers used in this study. [file Table_1.DOCX]

**Table S1.** Sequences of primers used in this study

|  | **Gene** | **Forward primer** | **Reverse primer** |
| --- | --- | --- | --- |
| qRT-PCR | *FON1* | ATCGACGCTTACAACAACAACC | CCGTCGTACTGGTTGTAGTAC |
|  | *FON2/FON4* | GTGTGTTTGCTTGACATGGG | CTCTGTCACGAGATCAAAAGA |
|  | *OsIG1* | AACGTGACCAAGCTGCTGAA | ATCGAGTGCGGCCCGTTATT |
|  | *RCN4* | GAGGTCATCGACAACTTCAA | CAGGAATATCAGTGACGATC |
|  | *REP1* | TACTACTACGAGCTCGCCGA | AGAAGCGCTGAAATGCTGGG |
|  | *Ubiquitin1* | AACAGCTGAGGCCCAAGA | ACGATTGATTTAACCAGTCCATGA |
|  | *OsMADS14* | ATGGGACCAGACACAACCTC | AAGCCTCCTTAGCCGTTGAT |
|  | *OsMADS15* | AACAAGGCTCTGCAGAAGGA | CATCATTTCTCTCGCCCATC |
|  | *OsMADS2* | GAAGACGAGAACAAGCTGCT | TTCAATCCAGTGGTGGATCA |
|  | *OsMADS4* | CAATCTGCGGGACAAGATG | GAGCTCCAGCTCCCTTATGC |
|  | *OsMADS16* | GGCAGGTGACCTACTCGAAG | GCATCGACATTTTGCTCAAG |
|  | *OsMADS3* | CAGCGTGATCAACCTGAAGA | CAACACACACAATATGGCAC |
|  | *OsMADS58* | GCAGGGGAAAGATTGAGATC | GTTGGTGATCTGTTGCTTCAG |
|  | *OsMADS13* | ATGGGGAGGGGCAGGATTGAG | TGCGCCTTCTTGTACCTGTCA |
|  | *OsMADS1* | ATCACCATCAGGGTCTTCTC | CAACCATGTCTGCTGCTTCA |
|  | *OsMADS5* | GCTTCATATATCTTGCCAAG | TTGGTTGAGGTGATCCATGT |
|  | *OsMADS7* | TGGGTTCTTCCATCCACTTG | CGTCATCATCATGGTAGCCA |
|  | *OsMADS8* | CACCTTGCAGATCGGGTTTA | ATCTGTGTCGTCACATCCGT |
|  | *OsMADS34* | CAACCAGAGCACTTCTTCCA | CTGAAGCTGAAACGGTAGCT |
|  | *OsMADS6* | AGAGAAAGACGCAACTGATGATGG | AGGCTTGCTGCATGGCTCTG |
|  | *LOC_Os01g41290* | TGAAGCCAAGTTGCTGCCTA | CATTCCATTCGCGACCACGA |
|  | *LOC_Os01g60730* | CCTCATCTCATCTCCCGAAA | CAGCTCATCGACTTCGTGTG |
|  | *LOC_Os02g01520* | TCCACACTGCCATCAGCCAT | GAAGAGGTCAAGGTCGAGGT |
|  | *LOC_Os07g29330* | CGCTCTACTTGGGGAACTTG | GTTGCATCAGCTGGAATCTG |
|  | *LOC_Os08g24880* | AGCGTATCGAAGCTTGGAGG | GGGAATAACTCTTCCGCAAG |
|  | *LOC_Os11g36160* | CAGTCAACAAGCTCGATGGG | ATCTGGAGTCTGAACCCAAC |
|  | *LOC_Os12g06080* | CCCTGCCAATTCATATGCCT | TTTGGCACTGTAGACACTGC |
|  | *LOC_Os12g41350* | GAAGCTATCGTTCTACGAGG | TTGGGAGCTGAAGTGTAACC |
| RT-PCR | *Ubiquitin1* | TGAAGACCCTGACTGGGAAG | CACGGTTCAACAACATCCAG |
|  | *OsVIL2* | GTGCCATCTTGAATGTGCTC | CATCCGTGAACGTTGAAATT |
| Cloning | *OsVIL2*_GUS | GGTACCTGACACTCTAAGGACTGCTC | GTGGATCCATGGCGAATTGG |
